# Raw Material Regulates Flavor Formation via Driving Microbiota in Chinese Liquor Fermentation

**DOI:** 10.3389/fmicb.2019.01520

**Published:** 2019-07-04

**Authors:** Chongchong Liu, Shengbao Feng, Qun Wu, Heqiang Huang, Zhanxiu Chen, Shanwen Li, Yan Xu

**Affiliations:** ^1^Key Laboratory of Industrial Biotechnology of Ministry of Education, State Key Laboratory of Food Science and Technology, School of Biotechnology, Jiangnan University, Wuxi, China; ^2^Suqian Industrial Technology Research Institute, Jiangnan University, Suqian, China; ^3^Qinghai Huzhu Barley Wine Co., Ltd., Haidong, China

**Keywords:** Chinese liquor, flavor producers, fructose, glucose, hulless barley

## Abstract

Raw material is important for flavors in fermented foods. Here, the effect of hulless barley on the microbiota in Chinese liquor was studied using two main cultivars (*heilaoya* and *dulihuang*). Six genera (*Lactobacillus, Saccharomyces, Komagataella, Aspergillus, Pichia*, and *Weissella*) were identified as flavor producers. *Komagataella*, mainly correlated with esters, dominated in *heilaoya*, and *Pichia*, mainly correlated with carbonyls, dominated in *dulihuang*. The Mantel test indicated reducing sugar drove the succession of microbiota (*heilaoya*: *P* = 0.001; *dulihuang*: *P* = 0.006). Especially, glucose (*P* = 0.0226) and fructose (*P* = 0.0168) presented the most significant correlations with *Pichia* and *Komagataella*, respectively. The simulative fermentation confirmed *Komagataella phaffii* QK2 grew better in *heilaoya* with more fructose, whereas *Pichia fermentans* PF grew better in *dulihuang* with more glucose. This work highlighted the effect of raw material on microbiota, which would be beneficial for regulating the quality of fermented foods.

## Introduction

Microbiota of spontaneously fermented foods have provided humans with tools for storing and flavor development for thousands of years (Giraffa, 2004; Wolfe and Dutton, 2015). The flavors metabolism and accumulation of fermented foods are driven by microbiota succession and their inter-related metabolic roles (Sulaiman et al., 2014), which determines the quality of fermented foods (Rhee et al., 2011). Dynamics and diversity of microbiota succession during the fermentation process are helpful for revealing the link between individual species and flavors (Walsh et al., 2016). Recent studies have focused on revealing how environmental factors affected microbiota succession during the fermentation process, such as moisture (Wolfe et al., 2014), temperature (De Filippis et al., 2016), pH (Tao et al., 2014), salt concentration (Lee et al., 2017), metabolites (Zheng et al., 2018), microbial interaction (Olanbiwoninu and Odunfa, 2018), and initial microbe (Ponomarova et al., 2017). However, it is still unclear about the effect of raw material on microbiota in food fermentation.

Raw material is a key factor for the quality of fermented foods. A right selection of raw material is very important for improving the content of nutrients in fermented bran. Moreover, many organic substances of raw material can be metabolized to flavors (Zhao et al., 2017). For instance, goji rawmaterial played a primary role in flavor production, such as esters, benzenes, aldehydes/ketones, acids, alcohols and other volatiles (Yuan et al., 2016). Apart from these, raw material can regulate the growth and metabolism of microbes. Crude glycerol with corn steep liquor and mineral salts metabolized by *Pichia guilliermondii* results in a maximum concentration of lipid (Kumar et al., 2017). Rye bran as fermentation matrix boosts *in situ* dextran production by *Weissella confusa* compared to wheat bran (Kajala et al., 2016). Thus, it is necessary to reveal the effect of raw material on microbiota and the subsequent quality of fermented foods.

In this study, we took Chinese liquor brewed with hulless barley as the research object. Hulless barley including two main cultivars (*heilaoya* and *dulihuang*) is used as raw material in Chinese liquor in Qinghai-Tibet Plateau. It is well known that the hulless barley (*Hordeum vulgare* L. *var. nudum*), also called “Qingke” in Chinese, is the major grain for Tibetans and an important livestock feed in the Tibetan Plateau (Zeng et al., 2015). However, the effect of hulless barley on the quality of Chinese liquor remains unknown. This study dissects the effect of hulless barley on microbiota and flavor production in Chinese liquor fermentation, which is important to reveal the relationship between raw material and the quality of spontaneously fermented foods.

## Materials and Methods

### Sample Collection

The fermentation of hulless barley liquor is a typical spontaneous and solid-state fermentation process, which includes starter making and alcoholic fermentation in pits. A complete production process consisted of four fermentation batches. The fermentation of each batch lasted for about 30 days. For the first fermentation batch, a mixture of hulless barley (*heilaoya* or *dulihuang*) and water (1:1.1) was steamed for 30–40 min before being mixed with 10% volume of same starters. Following a solid-state fermentation process, samples (fermented grains) were collected at different fermentation times (day 0, 5, 10, 15, 20, and 30) from six separate fermentation pits (three pits for each type) in May 2017 from Qinghai Huzhu Barley Wine Co., Ltd., in Qinghai Province, China. A 200.0 g per sample were stored at -20°C before microbial population determination, physical and chemical parameter analyses, and flavors analysis. Another 200.0 g per sample were collected and immediately frozen in liquid nitrogen, then kept on dry ice and stored at -80°C before DNA extraction. The third 200.0 g per sample were stored at 4°C for strain isolation.

### Strains Isolation and Identification

Six strains *Lactobacillus acetotolerans* B1, *Weissella viridescens* W1, *Saccharomyces cerevisiae* QK1, *Komagataella phaffii* QK2, *Aspergillus niger* M1, and *Pichia fermentans* PF, were all isolated from the hulless barley liquor fermentation process. Fermented grains (5.0 g) were suspended in 100 mL of sterile physiological saline (0.85% NaCl) and shaken for 15 min at room temperature. For the isolation of *S. cerevisiae* QK1, *K. phaffii* QK2, and *P. fermentans* PF, 0.1 ml of each decimal dilution was spread in triplicate on Wallerstein laboratory nutrient agar (WL) plates. The single colonies were inoculated into yeast extract-peptone-dextrose (YPD) medium and incubated at 30°C for 24 h under aerobic conditions. For the isolation of *A. niger* M1, 0.1 ml of each decimal dilution was spread in triplicate on potato dextrose agar (PDA) plates. The single colonies were inoculated into PDA plates and incubated at 30°C for 1–3 days under aerobic conditions. For the isolation of *L. acetotolerans* B1 and *W. viridescens* W1, 0.1 ml of each decimal dilution was spread in triplicate on de Man-Rogosa-Sharpe (MRS) agar plates. The single colonies were inoculated into MRS medium and incubated at 37°C for 36 h under anaerobic conditions. The genomic DNA of the single isolated strains was extracted according to the instruction of the TIANamp DNA kit (Tiangen, Beijing, China). For bacteria, 16S rRNA genes were amplified using the universal primer sets 27F and 1492R as described previously (Rochelle et al., 1992). For fungi, 26S rDNA genes were amplified using the universal primer sets NL1 and NL4 (Riethmüller et al., 2002). DNA sequencing of the PCR products was conducted by Sangon Biotech (Shanghai, China). The resulting sequences were compared with those available in the GenBank database at the National Center for Biotechnology Information (NCBI) using the basic local alignment search tool (BLAST). After a BLAST search against the sequences, the results were used for the identification of isolates.

### Flavors Analysis

Fermented grain (5.0 g) was mixed with 20 mL of deionized water, ultrasonically treated at 0°C for 30 min, and then centrifuged at 4°C (8,000 × *g*) for 5 min. Eight milliliters of supernatant and 20 μL menthol (internal standard, 100 μg/mL) were placed into a 20-mL headspace vial with 3 g NaCl. Flavors were determined by HS-SPME-GC-MS (GC 6890N and MS 5975; Agilent Technologies, Santa Clara, CA, United States) on a DB-Wax column (30 m × 0.25 mm i.d., 0.25 μm film thickness; J&W Scientific, Folsom, CA, United States), according to the reported method (Gao et al., 2014).

### Total DNA Extraction, Qualification, and Sequencing

Total DNA was extracted from 5 g fermented grains via the E.Z.N.A. (easy nucleic acid isolation) soil DNA kit (Omega bio-tek, Norcross, GA, United States) according to the processing instructions. Bacterial communities were studied by amplifying the V3-V4 hypervariable region of the 16S rRNA gene with universal primer sets F338 and barcode-R806 (Soergel et al., 2012). Fungal communities were studied by amplifying the internal transcribed spacer (ITS) region with primers ITS1F and ITS2R (Ihrmark et al., 2012). These primers contained a set of barcode sequences unique to each sample.

The PCR products were purified following a previous method (Ren et al., 2015). The purified PCR products were carried out on a MiSeq benchtop sequencer for 250-bp paired-end sequencing (2 × 250 bp; Illumina, San Diego, CA, United States) at Beijing Auwigene Tech., Ltd. (Beijing, China). All the generated raw sequences were analyzed using Qiime pipeline (v1.8.0) for preprocessing, taxonomic assignment, and community structure comparison (Caporaso et al., 2010). Quality trimming was conducted by removing the sequences with quality scores <30. Only sequences >200 bp were chosen for further analysis. The sequences that did not perfectly match the PCR primer, had not assigned tags, or had an N base were removed. Chimera sequences were removed using the Uchime algorithm (Edgar et al., 2011). A distance matrix was calculated from the aligned sequences and operational taxonomic units (OTUs) with a 97% identity threshold were clustered by Qiime’s uclust pipeline (Edgar, 2010). A single representative sequence from each clustered OTU was used to align to the Greengenes database (v13.8) (DeSantis et al., 2006) and the UNITE fungal ITS database (v6.0) (Koljalg et al., 2005). All sequencing data has been submitted to the DNA Data Bank of Japan (DDBJ) under the accession number of DRA007445 and DRA007455.

### Analyses of Physical and Chemical Parameters

In the fermentation process, the temperature was monitored in real time by inserting three thermometers (1 m in depth) into fermented grains in each pit (2.6 × 2.4 × 1.9 m). The moisture content was measured by determining its weight loss after drying 10 g of sample at 105°C for 4 h to ensure constant weight. The acidity of fermented grains was analyzed by acid-base titration (Wang et al., 2017). The reducing sugar content was assessed by means of a colorimetric method using 3, 5-dinitrosalicylic acid (Kai et al., 2008). To analyze the ethanol and organic acid content in fermented grain samples, 20 mL of distilled water was added to 5 g samples and ultrasonically treated at 0°C for 30 min, and then centrifuged at 4°C (8,000 × *g*) for 5 min. The obtained supernatant was filtered through a 0.22 μm syringe filter (Nylon Acrodisc, Waters Co., Milford, MA, United States) prior to analysis. The ethanol content was detected via high-performance liquid chromatography (HPLC) (Agilent 1200 HPLC, Agilent Technologies, Santa Clara, CA, United States) with a column (Aminex HPX-87H, 300 × 7.8 mm, Bio-Rad, Hercules, CA, United States) and a refractive index detector (WGE, Germany), according to published report (Wu et al., 2013). The organic acid content was determined on a Waters Acquity ultraperformance liquid chromatography (UPLC) H-Class system (Waters, Milford, MA, United States) equipped with a quaternary solvent manager, an auto-sampler, a column compartment, a photodiode array, and an Acquity UPLC HSS T3 analytical column (100 × 2.1 mm i.d., 1.8 μm film thickness; Waters, Milford, MA, United States), according to a previous report (Tang et al., 2015).

### The Sugar Profiles Analysis in Hulless Barley and Fermented Grains

To analyze the sugar profiles in hulless barley and fermented grain samples, one gram crushed hulless barley cultivar was added to 10 mL of 75% ethanol and ultrasonically treated at 0°C for 30 min. For fermented grains, five gram samples were added to 20 mL of 75% ethanol, ultrasonically treated at 0°C for 30 min. All samples were kept at 4°C for 12 h. After centrifugation (8000 × *g* for 10 min), the supernatant was collected and kept at water bath (80°C) for 30 min to remove protein. After centrifugation (8000 × *g* for 10 min), 500 μL of supernatant was collected and filtered by a 0.45 μm syringe filter (Nylon Acrodisc, Waters Co., Milford, MA, United States) and added to a screw-cap vial for subsequent analysis (Sun et al., 2014). The disaccharide content was determined by HPLC (Agilent 1100 HPLC, Agilent Technologies, Santa Clara, CA, United States) with a ZORBAX Eclipse XDB-C18 HPLC column (250 × 4.6 mm i.d., 5 μm film thickness, Agilent Technologies, Santa Clara, CA, United States) and a diode array detector, based on published report (Sun et al., 2014). The monosaccharide content was determined in a Dionex ICS-5000 ion chromatograph (Dionex, Sunnyvale, CA, United States) with a CarboPac-PA20 guard column (4 × 50 mm i.d., Dionex Corporation, Sunnyvale, CA, United States), and a CarboPac-PA20 analytical column (4 × 250 mm i.d., Dionex Corporation, Sunnyvale, CA, United States) and a pulsed amperometric detector, according to the previous report (Mellado-Mojica and Lopez, 2012).

### Monoculture and Co-culture Experiments

Six strains *Lactobacillus acetotolerans* B1, *Weissella viridescens* W1, *Saccharomyces cerevisiae* QK1, *Komagataella phaffii* QK2, *Aspergillus niger* M1, and *Pichia fermentans* PF, were used for monoculture and co-culture experiments. In monoculture, the medium for fungi was yeast extract 10 g/L, peptone 20 g/L and different concentrations of glucose or fructose (5, 10, 20, 40, and 80 g/L). The medium for the bacteria was peptone 10 g/L, beef extract 10 g/L, yeast extract 5 g/L, diammonium hydrogen citrate 2 g/L, Tween 80 1 mL/L, sodium acetate 5 g/L, dipotassium hydrogen phosphate 2 g/L, magnesium sulfate 0.58 g/L, manganese sulfate, 0.25 g/L, and different concentrations of glucose or fructose (5, 10, 20, 40, and 80 g/L). The monoculture experiments were conducted in triplicate in 96 deep-well plates (volume is 2 mL) containing 1 mL medium. Six strains were separately inoculated with 1 × 10^6^ CFU/mL into the liquid medium and each experiment was static incubated at 30°C for 72 h. In co-culture, they were separately inoculated with 1 × 10^6^ CFU/g into 250 mL shaking flask filled up with the solid medium (two cultivars of hulless barley) for co-cultures. Each experiment was statically incubated at 30°C for 30 days. Samples were collected from each flask at day 0, 5, 10, 15, 20, and 30 to determine microbial enumeration using qPCR. The flavors in co-cultures at the end of fermentation were determined by GC–MS.

### Quantitative Real-Time PCR (qPCR)

The qPCR was performed on Real-Time PCR System (Applied Biosystems) with the mixture of 8.2 μL ddH_2_O, 10.0 μL SYBR Green Supermix (SYBR^®^Premix Ex Taq^TM^ II, Takara, Shanghai, China), 0.4 μL (20 μM) primers and 1.0 μL DNA templates. The information about primers is listed in Table 1. PCR program for *L. acetotolerans* B1 and *W. viridescens* W1 is as follows: 95°C for 10 min, followed by 40 cycles at 95°C for 5 s, 60°C for 30 s, and 72°C for 45 s with a final extension from 72°C to 95°C. PCR program for *S. cerevisiae* QK1, *K. phaffii* QK2, *P. fermentans* PF and *A. niger* M1 is as follows: preheating at 98°C for 3 min, 40 cycles of 98°C for 30 s, 55°C for 30 s, and an increase of 0.5°C every 5 s from 65°C to 95°C for melting curve analysis to confirm the specificity of the amplification.

**Table 1:** The information about primers for qPCR in this study.

Amplicon	Primer direction	Primer sequence	The calibration curves
QK1	F	GGACTCTGGACATGCAAGAT	*y* = -0.2885x + 11.972
	R	ATACCCTTCTTAACACCTGGC	*R*^2^ = 0.997
QK2	F	TCGTTCATGGCAAGTTTCCG	*y* = -0.4517x + 14.524
	R	TTGCGGAACCCTCTTGCTTA	*R*^2^ = 0.9967
PF	F	CGGTAGACCAAGACACC	*y* = -0.367x + 13.884
	R	TGTAGAAGGTGTGGTGC	*R*^2^ = 0.9988
M1	F	CACTAACACCCCCATCCAGG	*y* = -0.4337x + 15.359
	R	ATGGTGTGATCGGGATGTCG	*R*^2^ = 0.9944
B1	F	TTCCTCCTGTTGGCGTTT	*y* = -0.3043x + 11.285
	R	TGCTGAAATGGTGGCAATAC	*R*^2^ = 0.9996
W1	F	TTGAATGACCCACAAGCGGT	*y* = -0.3581x + 12.004
	R	ATCGCACCCTGATCGTCTTC	*R*^2^ = 0.9919

*x is the Ct value; y is the lg copies ⋅ g^-*1*^.*

### Data Analysis

The flavors were analyzed by principal component analysis (PCA) using SPSS Statistics 19.0 (IBM^®^SPSS^®^Statistics, NY, United States). To associate the microbiota and flavors, Pearson correlations coefficient (*r*) was calculated between the microbial genera and flavors, and *r* > 0.6 with statistically significant (*P* < 0.05) was considered as a robust correlation (De Pasquale et al., 2014). The correlation matrix was visualized using Cytoscape 3.4.0 (Shannon et al., 2003). The Mantel test was used to assess a Spearman’s correlation between entries of the dissimilarity matrix of microbiota succession and the distance matrix of physical and chemical parameters in R (version 3.2.4) via the vegan package (version 2.3-4) (Xiao et al., 2017). Redundancy analysis (RDA) was conducted based on sugar profiles and flavor-producing microbiota in fermented grains and the explanatory of physical and chemical parameters to microbiota was calculated via the vegan package (version 2.3-4) in R (version 3.2.4) (Wang et al., 2018). A paired-sample *t*-test was conducted to test the discrepancy of sugar profiles in different hulless barley and physical and chemical parameters in fermented grains.

## Results

### The Effect of Raw Material on Flavor Production During Hulless Barley Liquor Fermentation

**FIGURE 1:**
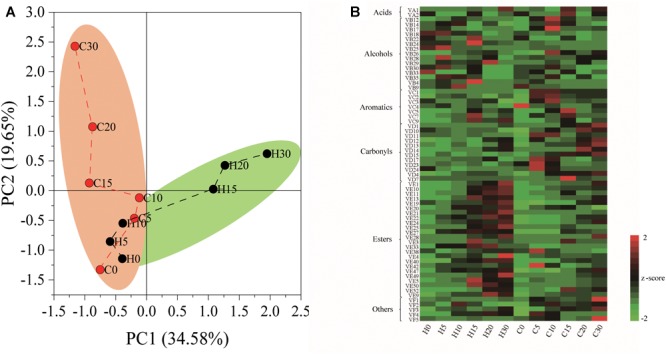
Flavors formation during fermentation. **(A)** Principal component analysis (PCA) based on flavors composition. **(B)** Heat map of flavors in fermented grains *heilaoya* and *dulihuang* during fermentation. A total of 65 flavors were identified from fermented grains, including 2 acids, 15 alcohols, 7 aromatics, 12 carbonyls, 24 esters, and 5 others. The Z score was used for data standardization. Fermentation time is shown as 0 to 30, e.g., “0” represents the sample fermented for day 0. H and C represents *heilaoya* and *dulihuang*, respectively.

The nutrient composition of hulless barley was detected, and the content of crude protein (157.5 mg/g) was significantly (*P* < 0.01) higher in *heilaoya*. However, the content of starch (588.9 mg/g) was significantly (*P* < 0.01) higher in *dulihuang*. To elucidate the effect of raw material on hulless barley fermentation, we processed two main cultivars of hulless barley fermentation. A total of 65 flavors were identified from fermented grains, including 2 acids, 15 alcohols, 7 aromatics, 12 carbonyls, 24 esters, and 5 others. PCA showed that the composition of flavors in fermented grains, *heilaoya* and *dulihuang*, were similar at the early stage of fermentation (day 0, 5, and 10), but differentiated after then (Figure 1A). The heat map revealed that more esters were produced in fermented grains *heilaoya* in the late stage of fermentation (day 15, 20, and 30), whereas the more carbonyls were produced in fermented grains *dulihuang* in the late stage of fermentation (day 20 and 30) (Figure 1B).

### Microbiota Pattern During Liquor Fermentation

**FIGURE 2:**
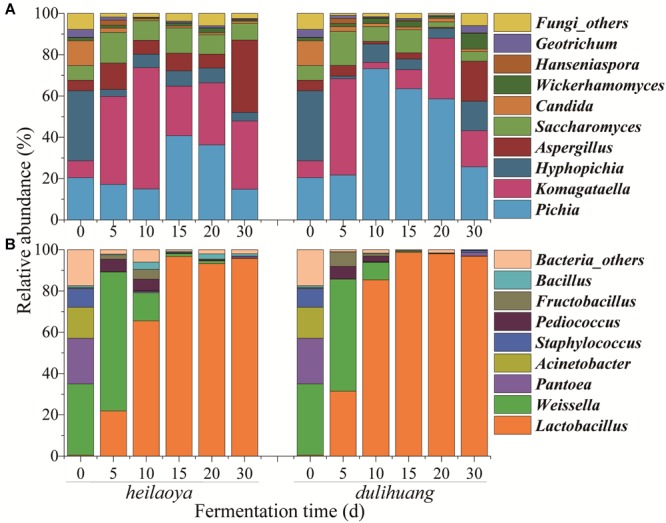
Distribution of microbiota during the fermentation process in two cultivars of hulless barley fermented grains (*heilaoya* and *dulihuang*). Temporal profile for the relative abundance of fungal taxa **(A)** and bacterial taxa **(B)** represents at the genus level. “Others” represents the genera with relative abundance <1%.

Temporal profile of microbiota structure in fermented grains of two cultivars of hulless barley were shown in Figure 2. A total of 56 fungal genera and 120 bacterial genera were identified in all fermented grains samples. Only 9 fungal genera and 8 bacterial genera were abundant (>1%). For fungal communities, *Pichia, Komagataella, Hyphopichia, Aspergillus, Saccharomyces, Candida, Wickerhamomyces, Hanseniaspora*, and *Geotrichum* were abundant in both fermented grains *heilaoya* and *dulihuang*. Although the fungal diversity showed no significant differences (*P* > 0.05) throughout the fermentation process, the relative abundances of *Pichia, Komagataella, Aspergillus*, and *Saccharomyces* were different in the two types of fermented grain (Figure 2A). *Pichia* dramatically increased from 22 to 73% in day 5 to day 10, and *Aspergillus* and *Saccharomyces* decreased from day 0 to day 20 in fermented grains *dulihuang*. Whereas, *Komagataella* dramatically increased from 8 to 59% before day 10, and *Aspergillus* and *Saccharomyces* were stable before day 20 in fermented grains *heilaoya* (Figure 2A). Thus, *Pichia* was more abundant in fermented grains *dulihuang* (*P* < 0.05) and *Komagataella* was more abundant in fermented grains *heilaoya* (*P* < 0.05).

For bacterial communities, the abundance of *Pantoea, Acinetobacter* and *Staphylococcus* all dramatically decreased, respectively, from the beginning, and was less than 2% at the end of fermentation in both fermented grains *heilaoya* and *dulihuang* (Figure 2B). Whereas, the abundance of *Weissella* significantly increased from 35 to 67% and 54% in the first 5 days, and dramatically decreased until the end of fermentation in both fermented grains *heilaoya* and *dulihuang*. Relative abundance of *Lactobacillus* significantly increased from 0.5 to 65% in the first 10 days in fermented grains *heilaoya*. Surprisingly, it dramatically increased to 85% in the first 10 days in fermented grains *dulihuang* (Figure 2B). It indicated that *Lactobacillus* increased faster in fermented grains *dulihuang* than *heilaoya*.

**FIGURE 3:**
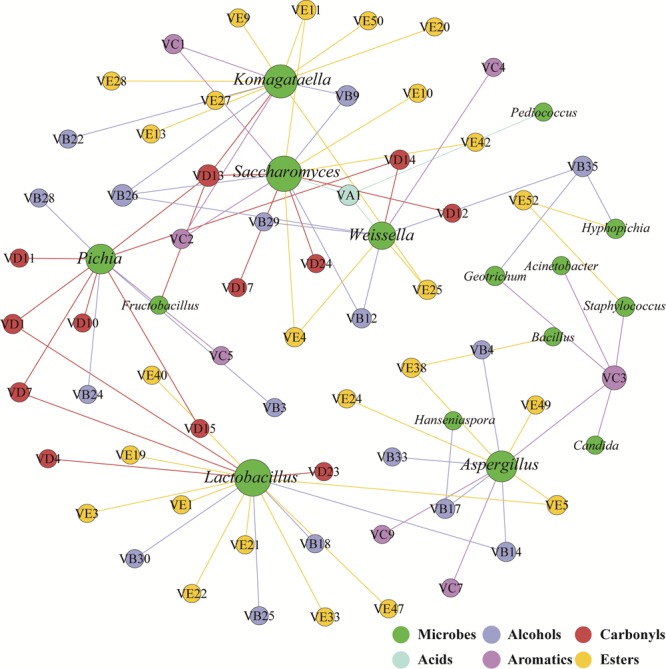
Correlation network between microbial genera (green) and flavors (other colored circles indicate different groups of compounds) was calculated by statistically significant (*P* < 0.05) and Pearson correlation coefficient (*r* > 0.6). The size of the circles is linked to the number of edge of microbial genera and flavors.

The correlation network between microbial genera and flavors was used to identify flavor-producing microbiota (Wang et al., 2016). *Lactobacillus, Saccharomyces, Komagataella, Aspergillus, Pichia*, and *Weissella* were positively associated with the flavors assigned to acids, alcohols, aromatics, carbonyls and esters (Figure 3). These six genera were defined as flavor-producing microbiota in hulless barley fermentation. And *Komagataella* was positively associated with many esters while *Pichia* was positively associated with many carbonyls (Supplementary Table S1).

### Relationships Between Microbiota and Physical and Chemical Parameters

The Mantel test was performed to assess significance of the Spearman’s correlation between the dynamics of physical and chemical parameters and the succession of the microbial community in fermented grains *heilaoya* and *dulihuang* (Table 2). Temperature, acidity, reducing sugar, ethanol, acetic acid and lactic acid were considered to be conventional endogenous factors during Chinese liquor fermentation (Song et al., 2017; Wang P. et al., 2018). And they were determined in fermented grains during hulless barley fermentation (Supplementary Figures S1, S2). In fermented grains *heilaoya*, reducing sugar (*r*^2^ = 0.6482, *P* = 0.001) and temperature (*r*^2^ = 0.4607, *P* = 0.043) correlated significantly with microbial community succession, while no significant correlation was shown between other parameters. In fermented grains *dulihuang*, only reducing sugar (*r*^2^ = 0.5788, *P* = 0.006) correlated significantly with microbial community succession (Table 2). Therefore, reducing sugar was determined to be an important diver of microbiota during hulless barley fermentation.

**Table 2:** Mantel test of physical and chemical parameters and microbial communities.

Mantel test	*Heilaoya*		*Dulihuang*	
	*r*^2^	*P*	*r*^2^	*P*
Reducing sugar	0.6482	0.001	0.5788	0.006
Temperature	0.4607	0.043	0.3713	0.061
Lactic acid	0.2942	0.094	0.2543	0.111
Acetic acid	0.3762	0.065	0.3234	0.074
Ethanol	0.1742	0.213	0.1255	0.341
Moisture	0.1887	0.118	0.2662	0.102
Acidity	0.3215	0.081	0.1687	0.231

*r^*2*^, Spearman correlation coefficient; *P* < 0.05 indicates a significant correlation.*

### Effect of Reducing Sugar on Flavor-Producing Microbiota

**FIGURE 4:**
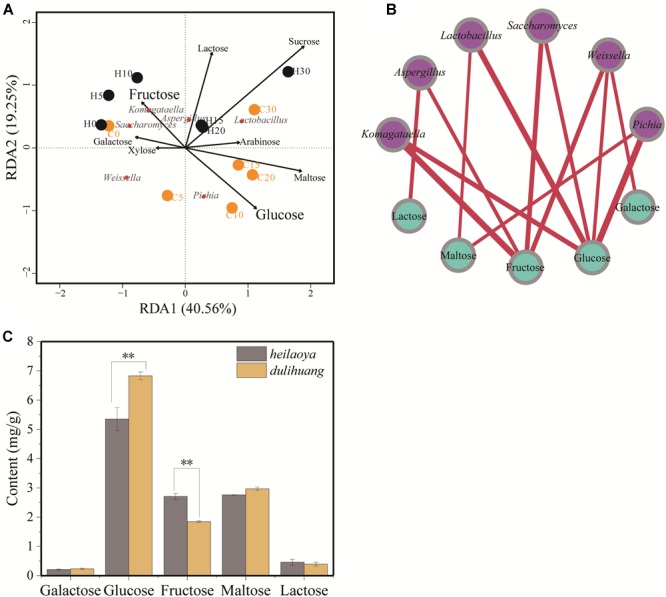
The composition of sugar profile and their association with flavor-producing microbiota succession. **(A)** Redundancy analysis (RDA) based on flavor-producing microbiota and sugar profiles in fermented grains. Fermentation time is shown as 0 to 30, e.g., “0” represents the sample fermented for day 0. H and C represents *heilaoya* and *dulihuang*, respectively. **(B)** Relationships between sugar profile and flavor-producing microbiota. A connection stands for a significant (*P* < 0.05) correlation. Size of each node is proportional to the number of connections, and the nodes are colored by blue (sugar) and purple (genus). The thickness of each connection between two nodes is proportional to the value of Spearman’s correlation coefficient (*ρ*). **(C)** Content of different sugars in hulless barley. Significant differences *P*< 0.01 (Students *t*-test) between *heilaoya* and *dulihuang* is denoted by ^∗∗^.

To explore the effect of reducing sugar on flavor-producing microbiota, the sugar profiles was determined during fermentation. Eight kinds of sugar (galactose, glucose, fructose, arabinose, xylose, sucrose, maltose, and lactose) were identified in fermented grains. The two axes explained 59.81% of the total variance in flavor-producing microbiota, suggesting a potential correlation between sugars and flavor-producing microbiota in fermented grains (Figure 4A). RDA revealed *Komagataella* was positively correlated with fructose (ρ = 0.0168) in fermented grains *heilaoya* and *Pichia* was positively correlated with glucose (ρ = 0.0226) in fermented grains *dulihuang*. It indicated that fructose and glucose in fermented grains presented significant correlation with flavor-producing microbiota (Figure 4B).

The sugar profiles in fermented grains were also monitored in the two cultivars of hulless barley (Figure 4C). Galactose, maltose, and lactose were not significantly different (*P*> 0.05). Whereas, the content of glucose was significantly higher in *dulihuang* (*P*< 0.01), and the content of fructose was significantly higher in *heilaoya* (*P*< 0.01). That suggested the sugar profiles in hulless barley selected flavor-producing microbiota in fermented grains, resulting in different flavors formation.

### Fructose and Glucose Drove the Flavor-Producing Microbiota

**FIGURE 5:**
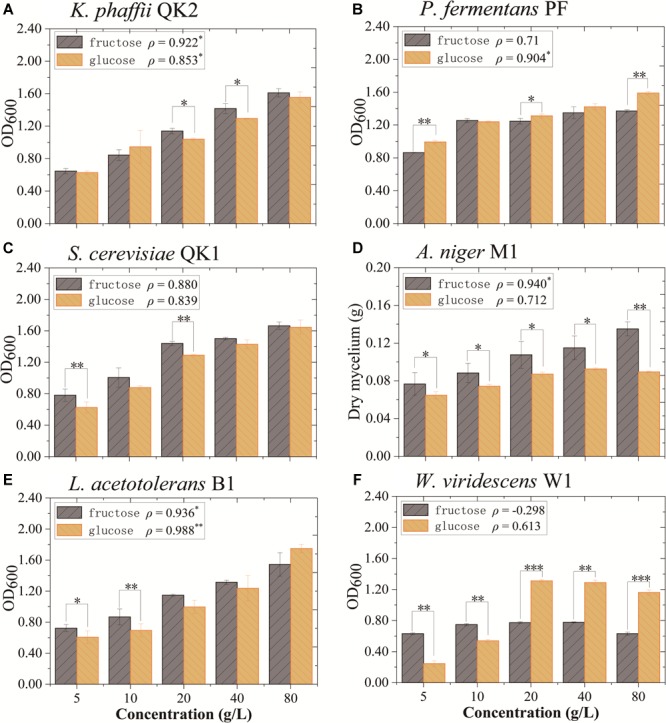
Growth of flavor-producing microbes in different concentrations of fructose and glucose after 3 days of cultivation. **(A)** *K. phaffii* QK2, **(B)** *P. fermentans* PF, **(C)** *S. cerevisiae* QK1, **(D)** *A. niger* M1, **(E)** *L. acetotolerans* B1, and **(F)** *W. viridescens* W1. The dry mycelium represents the biomass of *A. niger* M1 and the OD_600_ represents the biomass of other microbes. *ρ* indicates the value of Pearson correlation coefficient. Significant correlation (*P*< 0.05, *P*< 0.01, and *P*< 0.001) is denoted by ^∗, ∗∗^, and ^∗∗∗^.

The impact of fructose and glucose on flavor-producing microbiota was determined in monoculture experiments. Six strains (*L. acetotolerans* B1, *W. viridescens* W1, *S. cerevisiae* QK1, *K. phaffii* QK2, *A. niger* M1, and *P. fermentans* PF) isolated from this liquor fermentation process were used in the experiments. In monoculture, *K. phaffii* QK2 and *A. niger* M1 grew better in medium with fructose. They were positively correlated with fructose (*ρ* = 0.922^∗^ and *ρ* = 0.940^∗^) (Figure 5A,D). In contrast, *P. fermentans* PF was positively correlated with glucose (*ρ* = 0.904^∗^) (Figure 5B). *L. acetotolerans* B1 was both positively correlated with glucose and fructose (Figure 5E). But no significant correlation was shown for *S. cerevisiae* QK1 and *W. viridescens* W1 (Figure 5C,F). Therefore, *K. phaffii* QK2 and *A. niger* M1 showed preference for fructose, whereas *P. fermentans* PF showed preference for glucose.

### Solid State Fermentation of Flavor-Producing Microbiota

**FIGURE 6:**
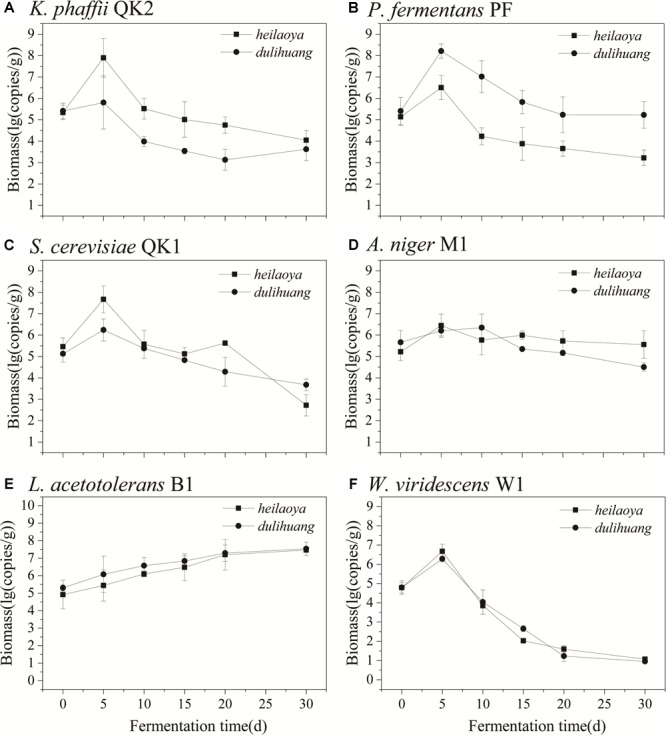
Comparison of biomass of flavor-producing microbes in co-cultures. **(A)** *K. phaffii* QK2, **(B)** *P. fermentans* PF, **(C)** *S. cerevisiae* QK1, **(D)** *A. niger* M1, **(E)** *L. acetotolerans* B1, and **(F)** *W. viridescens* W1.

**FIGURE 7:**
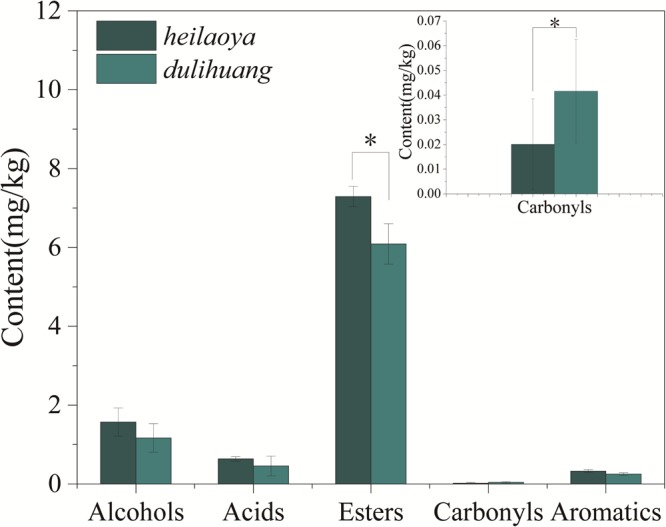
Content of different flavors at the end of fermentation. Significant differences *P*< 0.05 (Students *t*-test) is denoted by ^∗^.

The co-culture of six strains was performed in the solid medium with two cultivars of hulless barley as material separately. The cell growth of six strains in *dulihuang* was compared with those in *heilaoya*. *K. phaffii* QK2 was observed the significantly higher biomass (*P*< 0.05) in *heilaoya* throughout the fermentation process (Figure 6A) and *S. cerevisiae* QK1 had the highest biomass at day 5 in *heilaoya* (Figure 6C). However, *P. fermentans* PF and *L. acetotolerans* B1 had the significantly higher biomass (*P*< 0.05) in *dulihuang* throughout the fermentation process (Figure 6B,E). No significant differences for *A. niger* M1 and *W. viridescens* W1 were observed during the fermentation (Figure 6D,F). At the end of fermentation, the flavors profile of different fermented samples (*heilaoya* and *dulihuang*) was detected, including alcohols, acids, esters, carbonyls, and aromatics (Figure 7). The significantly higher content (*P*< 0.05) of esters (7.29 mg/kg) was found in *heilaoya*, and the significantly higher content (*P*< 0.05) of carbonyls (0.04 mg/kg) was discovered in *dulihuang*. These results showed *heilaoya* with higher fructose selected *K. phaffii* QK2, which produced more esters and *dulihuang* with higher glucose selected *P. fermentans* PF, which produced more carbonyls.

## Discussion

Raw material played an important role in providing various nutrients, precursors of flavor and epiphytic microbes to the food fermentation process (Gobbetti et al., 2014; Guan et al., 2018). This work showed an indirect contribution of raw material on food fermentation, in which the sugar profiles in hulless barley influenced flavor-producing microbiota and regulated flavors formation in Chinese liquor fermentation process.

In various fermented foods, microbiota were highly correlated with flavor production (Mota-Gutierrez et al., 2018; Wuyts et al., 2018). Six genera including *Lactobacillus, Saccharomyces, Komagataella, Aspergillus, Pichia*, and *Weissella* were identified as flavor-producing microbiota in hulless barley fermentation process in this work. Previous studies showed that, *Lactobacillus* led to producing 1-octen-3-ol, benzaldehyde, pentanal, octanal, nonanal, and 2-pentyl-furan in pork jerky fermentation (Zhao et al., 2016) and produced lactic acid and acetic acid in vegetable fermentation (Oguntoyinbo and Dodd, 2010) and yogurt (Pan et al., 2014). *Saccharomyces* produced large amounts of higher alcohols and acetate esters in food fermentation (Stribny et al., 2015). *Pichia* produced a total of 16 flavors, including 3-methylbutanal, 2-phenylethyl alcohol, phenylethyl acetate, 2,3-butadione in bread (Mo and Sung, 2014). These studies supported the contributions of *Lactobacillus, Saccharomyces*, and *Pichia* on flavors production in this work (Supplementary Table S1). And they were also reported as the core microbiota in other Chinese light aroma type liquor (Wang et al., 2019). *Aspergillus* was widely reported to produce many saccharifying enzymes to degrade the starch in raw material for food fermentation (Chen et al., 2014; Pervez et al., 2015). It would also interact with other microbes, such as *Lactobacillus* and *Saccharomyces*, hence regulate their flavor compounds producing (Ivey et al., 2013; Li et al., 2014). Very few studies showed the contribution of *Komagataella* and *Weissella* on flavors during food fermentation. Our work found *Komagataella* were positively correlated with 3-methyl-1-butanol, 1-butanol, pentanoic acid-ethyl ester, hexanoic acid-ethyl ester, and butanoic acid-3-methyl-ethyl ester. *Weissella* were positively correlated with pentanoic acid and 2-methoxy-5-methylphenol. These results suggested potential and important flavor-producing microbiota in Chinese liquor. Further studies are required to learn the contribution of these flavor-producing microbiota on flavors via monoculture and co-culture experiments with same raw material.

The composition and abundance of flavor-producing microbiota determine the flavor formation in liquor fermentation. In addition, some endogenous factors might strongly influence the composition and abundance of flavor-producing microbiota, such as moisture, temperature and pH (Tao et al., 2014; Wolfe et al., 2014; De Filippis et al., 2016). It is important to identify the main driving force to regulate microbiota during fermentation. In this study, we showed that reducing sugar was a key factor to influence microbial composition in Chinese liquor fermentation. Interestingly, a noteworthy increase of reducing sugar contents was observed at an early stage during fermentation process (Supplementary Figure S2A), which highlighted the intense hydrolyzing activity of microbial community, and the rate of hydrolysis of substrates to reducing sugar is faster than the utilization of reducing sugar (Sulaiman et al., 2014). An increase in reducing sugar was shown to be correlated with the increasing temperature and moisture, which could be taken as persuasive evidence of microbial growth and metabolism (Hubbe et al., 2010). This work highlighted that reducing sugar, as an important driver, affected the relative abundance of flavor-producing microbiota.

Notably, the sugar profiles could drive the growth and composition of microbiota during fermentation (Chatellard et al., 2016; Li et al., 2017; Lu et al., 2017). For bacterial community during *doubanjiang-meju* fermentation, *Pseudomonas* preferred glucose and arabinose while *Streptococcus* preferred arabinose, ribose and fructose, which resulted in bacterial succession and dynamic changes in organic acids and amino acids (Li et al., 2017). In addition, xylose, arabinose, glucose and cellobiose showed a strong selection for Clostridiaceae, Bacillaceae and Peanibacillaceae families in dark fermentation (Chatellard et al., 2016). In fructose-rich niches, the optimum substrate for the growth of fructophilic LAB was fructose, and its growth on glucose was very poor (Endo et al., 2009). Interestingly, in our study, we found that *Komagataella phaffii* QK2 grew better in *heilaoya* with more fructose, whereas *Pichia fermentans* PF grew better in *dulihuang* with more glucose. And the effects of hulless barley on glucose and fructose mainly included two points. On the one hand, hulless barley contained rich fructose and glucose (Figure 4C). On the other hand, the difference of nutrients would influence metabolism and enzyme activity of microorganisms (Singh et al., 2015; Zhu et al., 2017), especially glycosidase and polysaccharide hydrolase, which would influence synthesis and consumption of glucose and fructose. Knowing the selection of a particular sugar for microbiota would be beneficial for designing more efficient flavor-producing biological systems. Further studies should focus on the mechanism of the effect of these sugars on shaping the flavor-producing microbiota in Chinese liquor.

This work demonstrated that fructose and glucose of hulless barley affected the relative abundance of flavor-producing microbiota, hence influenced the flavor formation in Chinese liquor fermentation. It shed new light on the effect of raw material on food fermentation, and would contribute to improve the flavor of fermented foods by regulating the cultivar and composition of raw materials.

## Data Availability

The datasets generated for this study are available in the DNA Data Bank of Japan, DRA007445 and DRA007455.

## Author Contributions

CL, QW, and YX designed the study. CL, SF, QW, HH, ZC, SL, and YX performed the experiments and analyzed the data. CL wrote the manuscript.

## Conflict of Interest Statement

SF, HH, ZC, and SL were employed by the Qinghai Huzhu Barley Wine Co., Ltd. The remaining authors declare thatthe research was conducted in the absence of any commercial or financial relationships that could be construed as a potential conflict of interest.
