# Supplementary material for: Raw Material Regulates Flavor Formation via Driving Microbiota in Chinese Liquor Fermentation
Source: Front Microbiol. 2019 Jul 4;10:1520. doi: 10.3389/fmicb.2019.01520 (PMC6620735; doi:10.3389/fmicb.2019.01520)
Supplement: Supplementary file 1 [file Table_1.DOCX]

Supplementary Material

Raw material regulates flavor formation via driving microbiota in Chinese liquor fermentation

**Chongchong Liu^1^, Shengbao Feng^2^, Qun Wu^1*^, Heqiang Huang^2^, Zhanxiu Chen^2^, Shanwen Li^2^, Yan Xu^1*^**

^1^ Key Laboratory of Industrial Biotechnology of Ministry of Education; State Key Laboratory of Food Science and Technology, School of Biotechnology, Jiangnan University, Wuxi, Jiangsu, China; Suqian Industrial Technology Research Institute of Jiangnan University, Suqian, Jiangsu, China

^2^ Qinghai Huzhu Barley Wine Co. Ltd., Qinghai, China

*** Correspondence:**Corresponding Author: Qun Wu, Yan Xu
wuq@jiangnan.edu.cn, yxu@jiangnan.edu.cn

**This supplementary files includes:**

Supplementary Figures (Figure S1-S2)

Supplementary Tables (Table S1)

**Figure captions**

**Supplementary Figure S1.** The changes of temperature (A), acidity (B) and moisture (C) during fermentation process.

**Supplementary Figure S2.** The changes of contents of reducing sugar (A), ethanol (B), acetic acid (C) and lactic acid (D) during fermentation process.

**Supplementary Table S1.** Detailed information about the association between microbial genera and flavors. *r* represents Pearson correlation coefficient.

| Genus | *r* | ID | Compounds | Type |
| --- | --- | --- | --- | --- |
| *Saccharomyces* | 0.617 | VA1 | Pentanoic acid | acids |
| *Pichia* | 0.646 | VA1 | Pentanoic acid | acids |
| *Weissella* | 0.735 | VA1 | Pentanoic acid | acids |
| *Pichia* | 0.684 | VB33 | 1-Undecanol | alcohols |
| *Saccharomyces* | 0.694 | VB12 | 6-Methyl-5-hepten-2-ol | alcohols |
| *Saccharomyces* | 0.608 | VB26 | 2-Methyl-1-propanol | alcohols |
| *Weissella* | 0.699 | VB12 | 6-Methyl-5-hepten-2-ol | alcohols |
| *Hyphopichia* | 0.678 | VB35 | (S)-(+)-2-Heptanol | alcohols |
| *Saccharomyces* | 0.659 | VB9 | 3-Methyl-1-butanol | alcohols |
| *Aspergillus* | 0.641 | VB14 | 6-Undecanol | alcohols |
| *Weissella* | 0.623 | VB35 | (S)-(+)-2-Heptanol | alcohols |
| *Aspergillus* | 0.617 | VB4 | 2,6-Dimethyl-4-heptanol | alcohols |
| *Komagataella* | 0.685 | VB9 | 3-Methyl-1-butanol | alcohols |
| *Geotrichum* | 0.681 | VB35 | (S)-(+)-2-Heptanol | alcohols |
| *Komagataella* | 0.673 | VB22 | 1-Butanol | alcohols |
| *Pichia* | 0.654 | VB17 | (R)-(-)-2-Octanol | alcohols |
| *Lactobacillus* | 0.654 | VB14 | 6-Undecanol | alcohols |
| *Saccharomyces* | 0.647 | VB9 | 3-Methyl-1-butanol | alcohols |
| *Saccharomyces* | 0.745 | VB26 | 2-Methyl-1-propanol | alcohols |
| *Pichia* | 0.744 | VB24 | Tetrahydro-2-methyl-2-furanol | alcohols |
| *Komagataella* | 0.739 | VB26 | 2-Methyl-1-propanol | alcohols |
| *Pichia* | 0.738 | VB3 | 2-Ethyl-1-hexanol | alcohols |
| *Pichia* | 0.736 | VB28 | 4-Methyl-1-hexanol | alcohols |
| *Hanseniaspora* | 0.725 | VB17 | (R)-(-)-2-Octanol | alcohols |
| *Lactobacillus* | 0.72 | VB30 | 2-Furanmethanol | alcohols |
| *Lactobacillus* | 0.616 | VB25 | Linalool | alcohols |
| *Weissella* | 0.607 | VB26 | 2-Methyl-1-propanol | alcohols |
| *Lactobacillus* | 0.602 | VB18 | Phenylethyl alcohol | alcohols |
| *Pichia* | 0.838 | VC9 | p-Cresol | aromatics |
| *Weissella* | 0.696 | VC4 | 2-Methoxy-5-methylphenol | aromatics |
| *Pichia* | 0.683 | VC7 | 2-Methoxy-4-methylphenol | aromatics |
| *Pichia* | 0.718 | VC5 | 4-Ethyl-2-methoxyphenol | aromatics |
| *Aspergillus* | 0.685 | VD15 | Furfural | carbonyls |
| *Saccharomyces* | 0.699 | VD12 | Hexanal | carbonyls |
| *Pediococcus* | 0.624 | VD13 | Acetaldehyde | carbonyls |
| *Saccharomyces* | 0.697 | VD24 | 2-Octenal | carbonyls |
| *Komagataella* | 0.691 | VD13 | Acetaldehyde | carbonyls |
| *Aspergillus* | 0.687 | VD14 | 2-Methyl-1-butanal | carbonyls |
| *Lactobacillus* | 0.678 | VD7 | Benzeneacetaldehyde | carbonyls |
| *Lactobacillus* | 0.668 | VD4 | 2-Heptanone | carbonyls |
| *Pichia* | 0.725 | VD10 | 2,5-Dimethylbenzaldehyde | carbonyls |
| *Saccharomyces* | 0.725 | VD13 | Acetaldehyde | carbonyls |
| *Weissella* | 0.724 | VD14 | 2-Methyl-1-butanal | carbonyls |
| *Fructobacillus* | 0.718 | VD13 | Acetaldehyde | carbonyls |
| *Aspergillus* | 0.609 | VD7 | Benzeneacetaldehyde | carbonyls |
| *Komagataella* | 0.693 | VE27 | Pentanoic acid, ethyl ester | esters |
| *Saccharomyces* | 0.68 | VE4 | Propanoic acid, ethyl ester | esters |
| *Bacillus* | 0.621 | VE38 | Butanoic acid, 2-methyl-, ethyl ester | esters |
| *Lactobacillus* | 0.6 | VE19 | Acetic acid, hexyl ester | esters |
| *Weissella* | 0.682 | VE25 | Methyl 2-methylhexanoate | esters |
| *Lactobacillus* | 0.677 | VE21 | Isoamyl lactate | esters |
| *Komagataella* | 0.675 | VE13 | Hexanoic acid, ethyl ester | esters |
| *Lactobacillus* | 0.671 | VE5 | Butanoic acid, ethyl ester | esters |
| *Komagataella* | 0.655 | VE25 | Methyl 2-methylhexanoate | esters |
| *Lactobacillus* | 0.646 | VE40 | Propanoic acid, 2-hydroxy-2-methyl-, ethyl ester | esters |
| *Komagataella* | 0.743 | VE9 | Butanoic acid, 3-methyl-, ethyl ester | esters |
| *Komagataella* | 0.742 | VE28 | 1,1-Ethanediol, diacetate | esters |
| *Lactobacillus* | 0.734 | VE33 | 1-Butanol, 2-methyl-, acetate | esters |
| *Saccharomyces* | 0.732 | VE10 | Isocyanic acid, ethyl ester | esters |
| *Aspergillus* | 0.729 | VE38 | Butanoic acid, 2-methyl-, ethyl ester | esters |
| *Weissella* | 0.729 | VE4 | Propanoic acid, ethyl ester | esters |
| *Aspergillus* | 0.727 | VE5 | Butanoic acid, ethyl ester | esters |
| *Lactobacillus* | 0.725 | VE1 | Phenylacetic acid, ethyl ester | esters |
| *Saccharomyces* | 0.722 | VE42 | Propanoic acid, 2-methyl-, ethyl ester | esters |
| *Lactobacillus* | 0.719 | VE3 | Pentanoic acid, 2-hydroxy-4-methyl-, ethyl ester | esters |
| *Lactobacillus* | 0.602 | VE22 | Benzenepropanoic acid, ethyl ester | esters |

**Figure graphics**

**Supplementary Figure S1**

**Supplementary Figure S2**
